# Rapid, sensitive, and user-friendly detection of *Pseudomonas aeruginosa* using the RPA/CRISPR/Cas12a system

**DOI:** 10.1186/s12879-024-09348-3

**Published:** 2024-04-30

**Authors:** Wenjing Zhang, Hai Qu, Xin Wu, Jingjing Shi, Xinling Wang

**Affiliations:** 1 Medical college, Henan University of Chinese Medicine, No.156, Jinshui East Road, Zhengzhou, 450046 Henan China; 2 Autobio Diagnostics Co., Ltd., No.199, 15th Ave, Zhengzhou, 450016 Henan China; 3 Pharmacy College, Henan University of Chinese Medicine, No.156, Jinshui East Road, Zhengzhou, 450046 Henan China

**Keywords:** *Pseudomonas aeruginosa*, CRISPR/Cas12a, Recombinase polymerase amplification, Detection

## Abstract

**Background:**

*Pseudomonas aeruginosa* (*P. aeruginosa*) is a life-threatening bacterium known for its rapid development of antibiotic resistance, posing significant challenges in clinical treatment, biosecurity, food safety, and environmental monitoring. Early and accurate identification of *P. aeruginosa* is crucial for effective intervention.

**Methods:**

The *lasB* gene of *P. aeruginosa* was selected as the target for the detection. RPA primers for recombinase polymerase amplification (RPA) and crRNA for CRISPR/Cas12a detection were meticulously designed to target specific regions within the *lasB* gene. The specificity of the RPA/CRISPR/Cas12a detection platform was assessed using 15 strains. The detection limit of RPA/CRISPR/Cas12a detection platform was determined by utilizing a pseudo-dilution series of the *P. aeruginosa* DNA. The practical applicability of the RPA/CRISPR/Cas12a detection platform was validated by comparing it with qPCR on 150 samples (35 processed meat product samples, 55 cold seasoned vegetable dishes, 60 bottled water samples).

**Results:**

The RPA/CRISPR/Cas12a detection platform demonstrates high specificity, with no cross-reactivity with non-*P. aeruginosa* strains. This assay exhibits remarkable sensitivity, with a limit of detection (LOD) of 10^0^ copies/µL for fluorescence assay and 10^1^ copies/µL for the LFTS method. Furthermore, the performance of the RPA/CRISPR/Cas12a detection platform is comparable to that of the well-established qPCR method, while offering advantages such as shorter reaction time, simplified operation, and reduced equipment requirements.

**Conclusions:**

The RPA/CRISPR/Cas12a detection platform presents a straightforward, accurate, and sensitive approach for early *P. aeruginosa* detection and holds great promise for diverse applications requiring rapid and reliable identification.

**Supplementary Information:**

The online version contains supplementary material available at 10.1186/s12879-024-09348-3.

## Background

*Pseudomonas aeruginosa* (*P. aeruginosa*) belongs to the *Pseudomonadaceae* family and is a Gram-negative, aerobic, opportunistic pathogenic bacterium known for its remarkable adaptability and antibiotic resistance. It is ubiquitously found in various environments, encompassing both abiotic and biotic settings, including water, soil, animals, plants, as well as natural and artificial surroundings [1, 2]. *P. aeruginosa* wields an arsenal of virulence factors, such as endotoxin, exotoxin, proteolytic enzyme, and deploys diverse antimicrobial resistance mechanisms, including efflux pumps, antimicrobial-modifying enzymes, outer membrane permeability reduction, target modifications and more. Moreover, it exhibits the capacity to transition between different lifestyles, including planktonic, biofilm-based, and intracellular forms [3]. This adaptability enables it to activate, modify, and subvert host defense mechanism, evading immune system and antimicrobials agents, ultimately causing infections and diseases in both plants and animal hosts, including human [4]. In plants, *P.aeruginosa* can induce wet rot and leaf deformation [5], while in animals, it can lead to sepsis and respiratory infections, among other ailments [6]. In humans, *P. aeruginosa* poses a severe threat, particularly to individuals with compromised immune systems, such as those with severe burns, neutropenia, diabetes, organ transplants, cancer, and more. These infections are associated with substantial morbidity and mortality [7, 8]. Therefore, the early, rapid and accurate identification of *P. aeruginosa* is pivotal for effective clinical management, biosecurity, food safety, and environmental monitoring.

Currently, the detection of *P. aeruginosa* relies primarily on bacterial culture methods, immunological assays, and molecular analyses, among others. However, these methods exhibit certain limitations. Traditional bacterial culture methods, considered the gold standard for *P. aeruginosa* detection, are time-consuming, labor-intensive, and require strict conditions [9]. Immunological assays, reliant on antibodies, may yield false positives or negatives, undermining result accuracy. Molecular analyses, such as PCR-based methods, offer higher specificity and sensitivity for detecting *P. aeruginosa*. However, they require rigorous protocols, advanced equipment, and specialized training [10]. Consequently, there is an ongoing need to develop detection methods that are easier to operate and faster without compromising accuracy, to complement the existing molecular diagnostic techniques.

The clustered regularly interspaced short palindromic repeats (CRISPR) and the CRISPR - associated protein (CRISPR/Cas) system serves as an acquired immune system in bacteria and archaea, defending against mobile genetic elements invasions through rapid and precise identification and cleavage of specific nucleic acid sequences [11]. The CRISPR/Cas system has garnered significant attention in genetic engineering for its ability to swiftly, specifically, and efficiently recognize and cleave target nucleic acids [12]. In recent years, the CRISPR/Cas system, particularly utilizing Cas12 and Cas13 proteins with non-specific trans-cleavage activity, has emerged as a valuable tool in molecular detection, exemplified by platforms like SHERLOCK [13] and the HOLMES [14]. When the CRISPR/Cas system identifies and binds to the target sequences, it triggers the non-specific trans-cleavage activity of Cas12 or Cas13 proteins, leading to the cleavage of dual labeled single-stranded DNA (ssDNA) reporters, containing both a fluorescent maker and a quencher. This cleavage generates detectable or observable fluorescent signals [15]. Methods for detecting *P. aeruginosa* using the CRISPR/Cas12a system have been established by researchers [16–20]. Through a comprehensive review of the current literature, we have developed a rapid, accurate, and user-friendly diagnostic test for identifying *P. aeruginosa*, termed the RPA/CRISPR/Cas12a detection platform.

In this assay, we have identified the *lasB* gene as the target for detecting *P. aeruginosa* [21]. The *lasB* gene, acknowledged as a pivotal virulence factor contributing to disease pathology in patients, has demonstrated remarkable suitability for RPA amplification, with notable sensitivity in RPA detection [22–24]. Moreover, this gene is well-suited for assessing *P. aeruginosa* in clinical samples, as well as for monitoring the existence of this pathogen in drinking water, foodstuffs, and environmental samples [25].

Our detection platform combines the precision of the CRISPR/Cas12a mechanism with the efficiency of RPA isothermal amplification, thereby simplifying the detection process and significantly reducing the time required for precise pathogen identification. This assay’ s utility is especially advantageous in resource-limited settings, providing an effective means for early intervention and management of infections caused by *P. aeruginosa*.

Consequently, our research findings contribute to further enhancing the potential of the *lasB* gene for developing advanced diagnostic tools specifically targeting *P. aeruginosa*.

## Methods

### Reagents and instruments

**Fig. 1:**
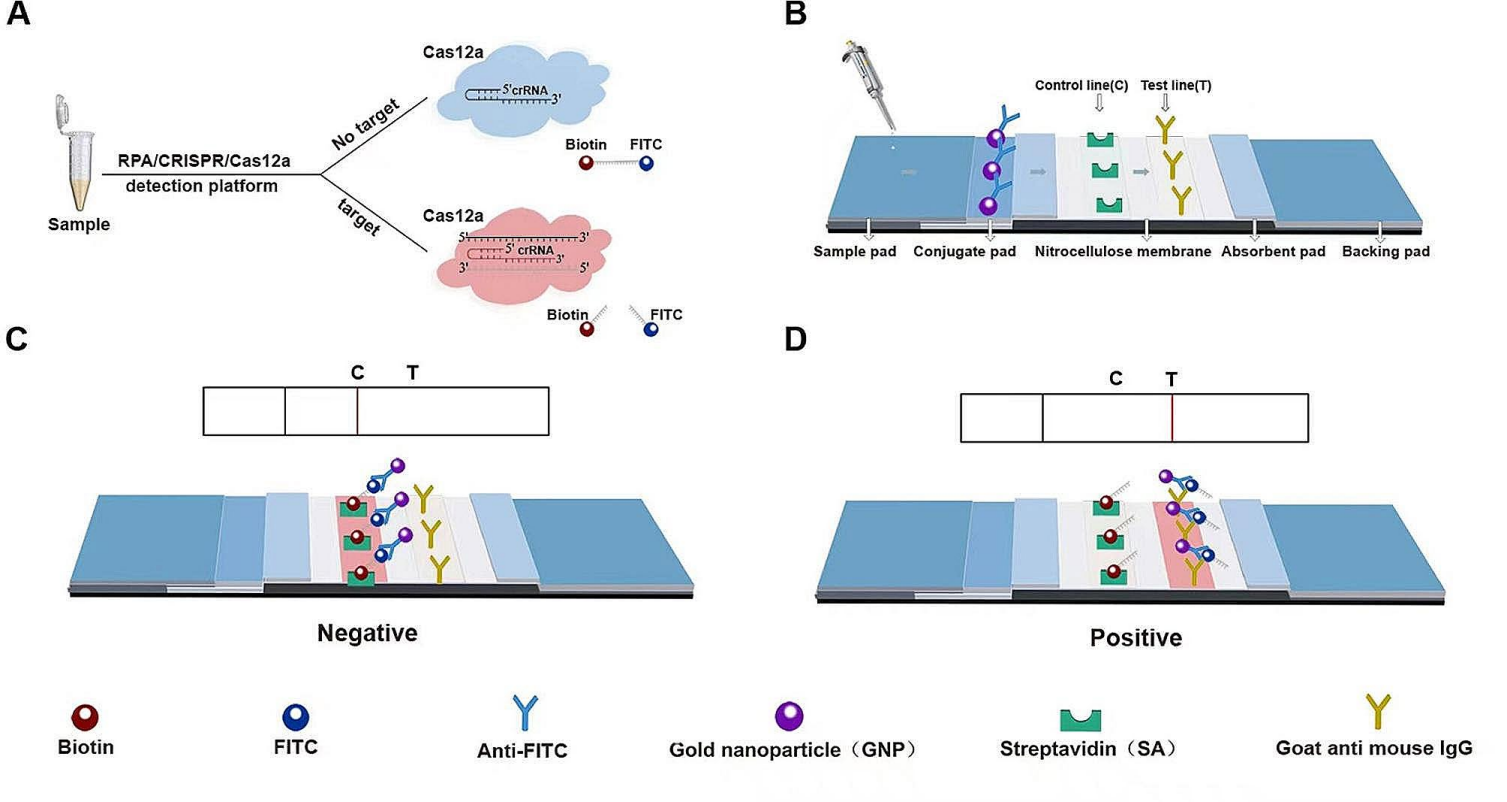
Schematic illustration of the principle of the lateral flow test strip. (**A**) Following RPA amplification, the samples undergo CRISPR/Cas12a cleavage reaction. (**B**) The test strip displays the positions of gold nanoparticles, anti-FITC antibody, streptavidin (SA), and goat anti-mouse IgG. (**C**) The complexes of gold nanoparticle-anti-FITC antibody-ssDNA reporter are captured by SA, resulting in the visibility of the C line. (**D**) In the presence of the target, the complexes of FITC-anti-FITC antibody-gold nanoparticle are captured by the goat anti-mouse IgG, thereby making the T line visible

The Isothermal DNA Amplification Basic Kit for RPA was sourced from EZassay Ltd. (Shenzhen, China). LbaCas12a enzyme and its corresponding reaction buffer were purchased from New England Biolabs (Ipswich, MA, USA). The TIANamp Bacteria DNA kit was sourced from Tiangen Biotech Co., Ltd (Beijing, China). Ultrapure water (dd H_2_O) was purchased from Thermo Fisher Scientific (Shanghai, China). RPA primers, crRNA and the ssDNA reporters (fluorescence assay: 5′ FAM - TTATT - BHQ1 3′, lateral flow test strip (LFTS) method: 5′ FITC - TTTTTTTTTT - Biotin 3′) were custom-synthesized by Sangon Biotech Co.,Ltd (Shanghai, China). The ABI 7500 fast system was purchased from Applied Biosystems (Foster City, CA, United States). Lateral flow strips were obtained from Tiosbio (Beijing, China) and their principle is illustrated in Fig. 1. The test strip’s conjugate pad was coated with complexes of gold nanoparticles and anti-FITC antibody. Streptavidin (SA) and goat anti-mouse IgG were immobilized on the NC membrane at the positions corresponding of the C line and the T line, respectively (Fig. 1B). In the absence of the target, the trans-cleavage activity of Cas12a remained inactive, keeping the ssDNA reporters intact (Fig. 1A). Complete ssDNA reporters, along with complexes of gold nanoparticle and anti-FITC antibody, were captured by SA on the C line, resulting in the visibility of the C line while the T line invisible (Fig. 1C). In the presence of the target, the trans-cleavage activity of Cas12a was activated, leading to the separation of FITC and biotin molecules within the ssDNA reporters. FITC-anti-FITC antibody-gold nanoparticle complexes were captured by the goat anti-mouse IgG on the T line, rendering the T line visible, with no change to the visibility of the C line (Fig. 1D).

### Bacteria culture and DNA extraction

A standard *P. aeruginosa* strain, four clinically isolated *P. aeruginosa* strains, and ten non - *P. aeruginosa* strains were generously provided by Autobio Diagnostics Co., Ltd. Detailed information on the strains used in this study is presented in Table 1. The bacterial strains were stored in Luria-Bertani (LB) broth supplemented with 20% glycerol at -70℃. When required, the preserved bacteria were streaked onto agar plates and incubated at 37℃ for 24 h. Single colonies were picked and further incubated at 37℃ for 24 h. The bacterial suspension was subsequently standardized to a final concentration of 1 × 10^5^ CFU/mL for subsequent experimental procedures. Genomic DNA was extracted from these bacterial cultures using the TIANamp Bacteria DNA kit for all experiments except sensitivity analysis, following the standard manufacturer’s instructions.

**Table 1:** Information of bacteria strains

Bacteria strain	Source
*P. aeruginosa*	ATCC27853
*P. aeruginosa*	ATTL2104080901
*P. aeruginosa*	ATTL2104080902
*P. aeruginosa*	ATTL2104080903
*P. aeruginosa*	*ATTL2104080904*
*P. stutzeri*	*CTCC23621*
*P. putida*	*CTCC20575*
*P. alcaligenes*	*CICC23927*
*P. fluorescens*	*CTCC23919*
*Staphylococcus aureus*	*ATCC29213*
*Escherichia coli*	*ATCC25922*
*Shigella flexner*	*ATCC12022*
*Listeria monocytogenes*	*ATCC19118*
*Salmonella typhimurium*	*ATCC14028*
*Bacillus subtilis*	*ATCC19659*

### RPA primer and crRNA design

**Fig. 2:**
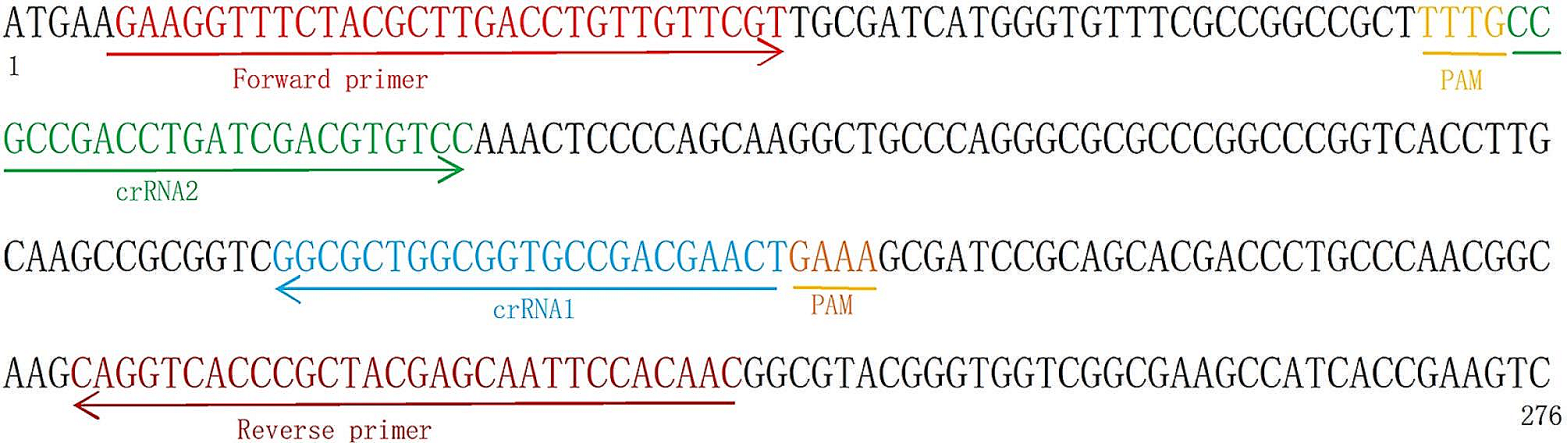
The design sites of the RPA primers and crRNA

For the detection of *P. aeruginosa*, the *lasB* gene was selected as the target due to its significant intraspecific conservation and notable interspecific similarity, as observed in the genome sequences of *P. aeruginosa* from GenBank. To ensure specificity, RPA primers and crRNA were meticulously designed to target specific regions within the *lasB* gene. The design locations for the RPA primers and crRNA are visually represented in Fig. 2, and the sequences of these primers and crRNA can be found in Table 2.

**Table 2:** The sequences of the primers and crRNA

Application	sequence
RPA primer	Forward 5′ GAAGGTTTCTACGCTTGACCTGTTGTTCGT 3′
	Reverse 5′ GTTGTGGAATTGCTCGTAGCGGGTGACCTG 3′
crRNA	crRNA1 5′ UAAUUUCUACUAAGUGUAGAUAGUUCGUCGGCACCGCCAGCGCC 3′
	crRNA2 5′ UAAUUUCUACUAAGUGUAGAUCCGCCGACCUGAUCGACGUGUCC 3′

### RPA reaction

The RPA amplification was conducted using the Isothermal DNA Amplification Basic Kit following the manufacturer’s standard instructions. The amplification system (20µL) comprised the following components: Rehydration Buffer (2X) 10µL, Forward Primer (20µM) 0.5µL, Reverse Primer (20µM) 0.5µL, DNA template 1µL, ddH_2_O 6µL, and Starter (10X) 2µL. The mixture was gently mixed by flicking and centrifuged at low speed for 10 s, repeat this step three times. The reaction system was then incubated at 39℃ for various duration, including 5 min, 10 min, 15 min, 20 min, 25 min, and 30 min, to determine the optimal incubation time. As a negative control, ddH_2_O was used.

### Screening of crRNA

To identify the crRNA for CRISPR/Cas12a detection, crRNA1 and crRNA2 were separately introduced into the CRISPR/Cas12a detection reaction system, followed by the observation of fluorescence intensity. The reaction system (40µL) consisted of the following components: 10µL reaction buffer (4X), 1µL Reporter (4µM, 5′ FAM – TTATT - BHQ1 3′), 2µL Cas12a Protein (1µM), 2µL crRNA (1µM), and 23µL ddH_2_O, pre-mixed accordingly. Subsequently, 2µL of RPA amplification products were added and thoroughly mixed. The reaction tube was immediately transferred to a Real-time fluorescence PCR instrument. The fluorescence resulting from the Cas12a cleavage reaction was continuously collected using the ABI fast 7500 system. Background-subtracted fluorescence was determined by subtracting the background fluorescence (without template) from the initial fluorescence of all samples. Subsequently, the background subtracted fluorescence intensities were compared under the same reaction conditions to select the optimal crRNA and reaction time based on the results obtained.

### CRISPR/Cas12a detection

Both fluorescence assay and the LFTS method were employed for the detection of the amplified *P. aeruginosa* DNA. In the fluorescence assay, the reaction system and specific operational steps were consistent with those used for crRNA screening, with the only difference being the use of the optimal crRNA. Real-time fluorescence values were collected using the ABI 7500 Fast system. Regarding the LFTS method, the reaction system was the same as that of the fluorescence assay, except that 5′ FITC - TTTTTTTTTT - Biotin 3′ was used instead of 5′ FAM - TTATT - BHQ1 3′ as the reporter. After thorough mixing of the mixture, the reaction incubated at 37℃ for the optimal reaction time selected. Subsequently, the reaction product was dripped onto the sample end of the test strip, followed by observation of the T line and C line.

### Evaluation of specificity of RPA/CRISPR/Cas12a detection platform

The specificity of the RPA/CRISPR/Cas12a detection platform was assessed using a range of strains as listed in Table 1. This included 1 standard strain of *P. aeruginosa*, 4 clinically isolated of *P. aeruginosa*, 4 *Pseudomonas* strains closely related to *P. aeruginosa*, and 6 non-*Pseudomonas* strains. Each strain was individually subjected to detection using the RPA/CRISPR/Cas12a platform at a concentration of 1 × 10^5^ CFU/mL. The assays were performed three times, following the methodology described above.

### Evaluation of the sensitivity of RPA/CRISPR/Cas12a detection platform

To assess the sensitivity of RPA/CRISPR/Cas12a detection platform, a recombinant plasmid containing the *lasB* target sequence (pGEM - T Easy - *lasB*) was subjected to continuous 10 - fold dilution, ranging from 10^6^ to 10^0^ copies/µL. Subsequently, the differently diluted DNA samples were used as templates to determine LOD. All assays were replicated 3 times using the aforementioned methodology.

### Validating the effectiveness of RPA/CRISPR/Cas12a detection platform with Food samples

To validate the practical applicability of the RPA/CRISPR/Cas12a detection platform, a total of 150 diverse food samples were analyzed, including processed meat products, cold seasoned vegetable dishes from nearby markets, and college-provided bottled water. Specific methods for sample processing are referenced in the literature [26]. Additionally, for a comprehensive comparison, these samples were concurrently subjected to detection using qPCR. In particular, qPCR was executed in a 25µL volume with 12.5µL of 2×SYBR Green PCR mix, 2µL of each primer at 1 µM concentration, 1µL of DNA template, and 7.5µL ddH_2_O. The PCR program began with a 10-minute denaturation at 95℃, followed by 40 cycles at 95℃ for 15 s for denaturation, and annealing/extension at 60℃ for 30 s, using primers described in prior literature [21]. Each assay was performed in triplicate, adhering strictly to the described protocol.

### Statistical analysis

Statistical analyses were performed using SPSS 21.0 (IBM SPSS 21.0, SPSS, Inc., Chicago, IL, USA). A Studentʼs *t* test was employed for the comparisons between two groups. All data were presented as mean ± SD. Statistical significance was set as *P* < 0.05. Each experiment was performed in triplicate.

## Results

### Optimal crRNA for CRISPR/Cas12a detection

**Fig. 3:**
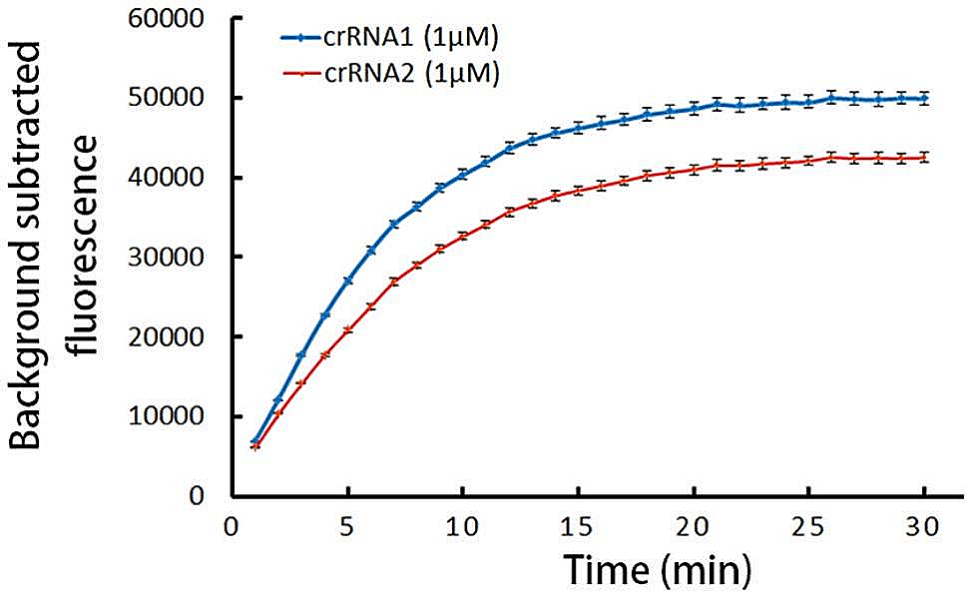
The fluorescence curves generated by CRISPR/Cas12a detection reaction. crRNA 1: the reaction system with crRNA1 addition, crRNA 2: the reaction system with crRNA2 addition

The RPA product, amplified at the optimal reaction time of 15 min, was employed as the template for screening crRNA in the CRISPR/Cas12a detection. crRNA1 and crRNA2 were separately introduced into the reaction system, and the background-subtracted fluorescence values were compared between the crRNA1 and the crRNA2 groups. As illustrated in Fig. 3, the fluorescence values of the crRNA1 group were higher than those of the crRNA2 group (*P* < 0.05), establishing crRNA1 as the optimal choice for CRISPR/Cas12a detection. Additionally, Fig. 3 demonstrates that the fluorescence values of the crRNA1 group were consistently higher than the crRNA2 group throughout the duration of the reaction. The fluorescence curve analysis further revealed that background-subtracted fluorescence reached a plateau after approximately 15 min, confirming 15 min as the optimal reaction time for the CRISPR/Cas12a detection method.

### Specificity of RPA/CRISPR/Cas12a detection platform

**Fig. 4:**
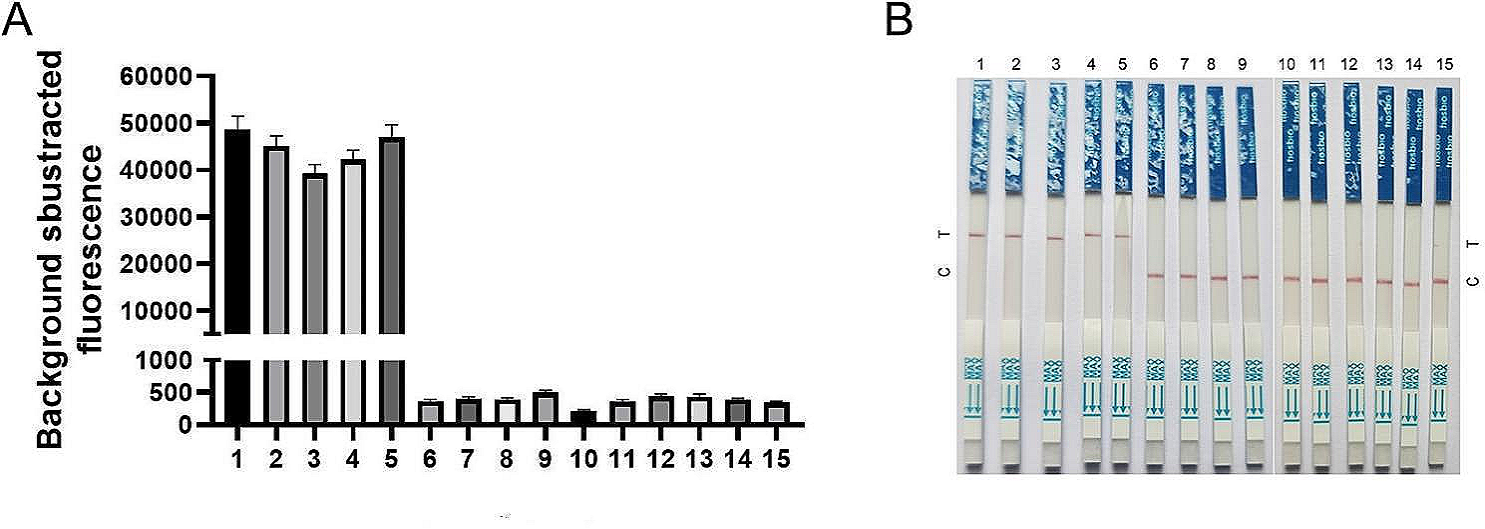
Specificity assay of the RPA/CRISPR/Cas12a detection platform. (**A**) Background - subtracted fluorescence intensity of fluorescence assay method, values represent the mean ± SD (*n* = 3). (**B**) The outcomes of the LFTS method. 1. *P. aeruginosa* standard strain, 2–5. *P. aeruginosa* clinically isolated strains, 6. *P. stutzeri*, 7. *P. putida*, 8. *P. alcaligenes*, 9. *P. fluorescens*, 10. *Staphylococcus aureus*, 11. *Escherichia coli*, 12. *Shigella flexner*, 13. *Listeria monocytogenes*,14. *Salmonella typhimurium*, 15. *Bacillus subtilis*

The RPA/CRISPR/Cas12a detection platform was employed to assess 15 bacteria strains (Table 1). The fluorescence assay results demonstrated that there was a significant increase in fluorescence values for the *P. aeruginosa* standard strain and four clinically isolated strains of *P. aeruginosa* (*P*<0.05), while fluorescence values remained relatively unchanged for other non-*P. aeruginosa* strains (Fig. 4A). The outcomes from the LFTS method were consistent with those of the fluorescence assay, showing positive results exclusively for the *P. aeruginosa* standard strain and 4 *P. aeruginosa* clinically isolated strains (Fig. 4B). These results confirmed the high specificity of the RPA/CRISPR/Cas12a detection platform in accurately identifying *P. aeruginosa*.

### Sensitivity of the RPA/CRISPR/Cas12a detection platform

**Fig. 5:**
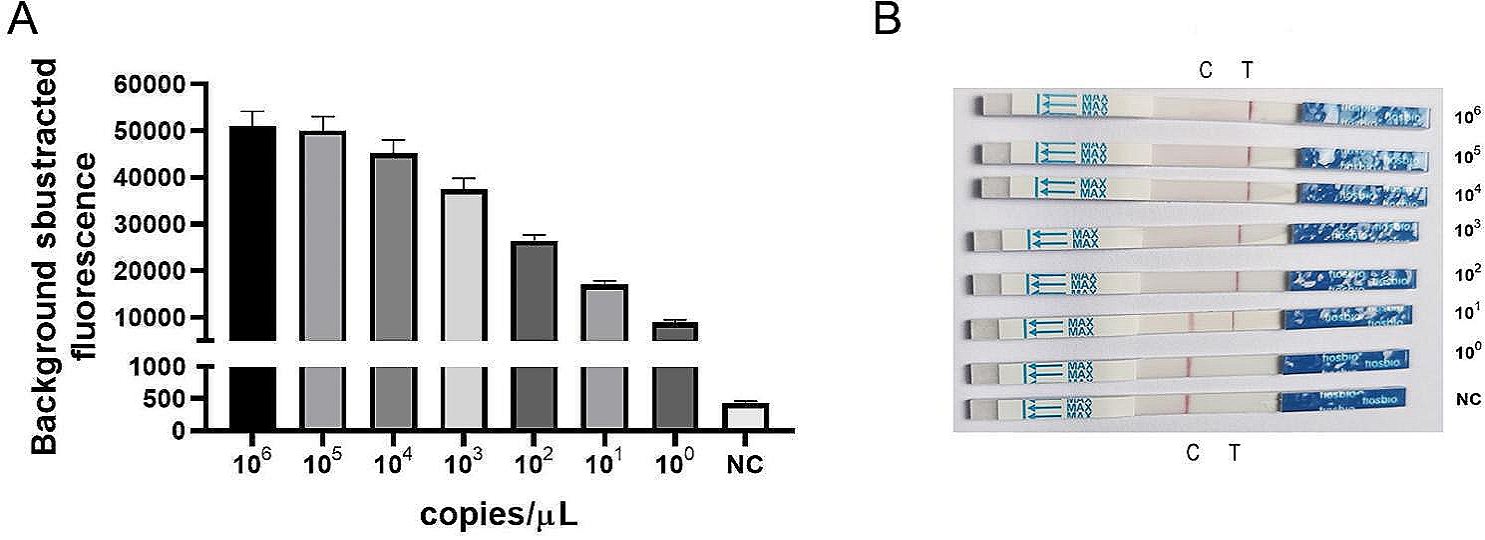
Sensitivity of the RPA/CRISPR/Cas12a detection platform (**A**) The histogram of background-subtracted fluorescence intensity obtained from different DNA concentration of *P. aeruginosa*, values represent the mean ± SD (*n* = 3). (**B**) The result of the LFTS method

To assess the sensitivity of the RPA/CRISPR/Cas12a detection platform, a recombinant plasmid containing the *lasB* target sequence (pGEM - T Easy - *lasB*) was subjected to serial 10-fold dilutions, ranging from 10^6^ to 10^0^ copies/µL. Each diluted sample was analyzed using the RPA/CRISPR/Cas12a detection platform to determine the LOD, with ddH_2_O serving as the negative control. As depicted in Fig. 5, a significant fluorescence signal was observed using fluorescence assay when the DNA concentration exceeded 10^0^ copies/µL (*P*<0.05). Interestingly, the results obtained from the LFTS method were generally consistent with those of the fluorescence assay.The appearance of T line was observed at a concentration of 10^1^ copies/µL and above. Among the positive results, both the T line and C line became visible at a concentration of 10^1^ copies/µL, it is speculated that the trans-cleavage activity of Cas12a was insufficiently activated due to the low DNA concentration, resulting in only a portion of ssDNA reporters were trans-cleaved. Consequently, the ssDNA reporter-gold nanoparticle-anti-FITC antibody complexes were captured on the C line, while the FITC-anti-FITC antibody-gold nanoparticle complexes were captured on the T line, making both the T line and C line visible. These results indicate that RPA/CRISPR/Cas12a detection platform exhibits high sensitivity, with a LOD of 10^0^ copies/µL for fluorescence assay and 10^1^ copies/µL for the LFTS method.

### Application of the RPA/CRISPR/Cas12a detection platform

To evaluate the applicability of RPA/CRISPR/Cas12a detection platform, a total of 150 samples were tested using both qPCR and the RPA/CRISPR/Cas12a detection platform. The results obtained from the fluorescence assay and the LFTS method were compared with the results of the qPCR assay. The findings are presented in Table 3, demonstrating that the results of both the fluorescence assay and the LFTS method were generally consistent with the qPCR assay. This suggests that the performance of the RPA/CRISPR/Cas12a detection platform is comparable to that of qPCR. One notable advantage of the RPA/CRISPR/Cas12a detection platform is its ability to deliver results in a shorter time and at isothermal temperature compared to qPCR. Consequently, the RPA/CRISPR/Cas12a detection platform offers a convenient and efficient alternative for pathogen detection.

**Table 3:** Comparison of RPA/CRISPR/Cas12a detection platform with qPCR

Sample	Detection method	Positive	Negative	Total	Detection rate(%)
Processed meat products	Fluorescence assay	4	31	35	11.43
	The LFTS method	3	32	35	8.57
	qPCR	4	31	35	11.43
Cold seasoned vegetable dishes	Fluorescence assay	9	46	55	16.36
	The LFTS method	9	46	55	16.36
	qPCR	10	45	55	18.18
Bottled water	Fluorescence assay	2	58	60	3.33
	The LFTS method	1	59	60	1.67
	qPCR	2	58	60	3.33

## Discussion

*P. aeruginosa*, renowned for its multidrug resistance and association with high mortality rate, has prompted extensive research on early and rapid detection methods. Among these methods, molecular analyses, especially PCR-based approaches, have gained popularity for their high sensitivity and specificity in detecting in *P. aeruginosa*. Various target genes have been employed in PCR-based methods for *P. aeruginosa* detection, including *lasB* gene [25], *gyrB* gene [27, 28], *oprl* gene [29, 30] *ecfX* gene [31, 32], among others. Through bioinformatics analysis, it was determined that the *lasB* gene exhibits high intraspecific conservation and significant interspecific dissimilarity, making it a promising target for detection. Furthermore, as a key virulence factor, the *lasB* gene has potential applications in anti-virulence treatments [21].

In this study, the selection of the *lasB* gene as the target for the RPA/CRISPR/Cas12a detection platform exhibited exceptional specificity. It successfully identified both the standard strain and clinically isolated strains of *P. aeruginosa* without cross-reactivity to non - *P. aeruginosa* strains. This outcome is consistent with the findings reported by Yang et al. [20], aligning with the bioinformatics analysis. Additionally, the platform exhibited excellent sensitivity, with LOD of 10^0^ copies/µL and 10^1^ copies/µL for the fluorescence assay and LFTS method, respectively. This is in line with the findings of Yang et al. [20], who identified an LOD of 15.9 CFU/reaction in their methodology, further validating suitability of the *lasB* gene as a target for *P. aeruginosa* detection.

The RPA/CRISPR/Cas12a detection platform employs RPA for isothermal DNA amplification of *P. aeruginosa* DNA, offering a user-friendly operation. The designed primers allowed the amplification product to reach a plateau within 15 min, indicating the detection platform’s rapidity and efficiency. By utilizing crRNA1 in the CRISPR/Cas12a detection, the fluorescence signal can reach approximately 80% of the maximum value within 10 min, allowing the platform to yield results in a remarkably short period of approximately 15 min. Combined with the RPA reaction duration, the entire detection process of the RPA/CRISPR/Cas12a detection platform can be completed in approximately 30 min. Moreover, the LFTS method is notably less complex and easier to operate, and it generally results in shorter testing times.

In practical sample evaluation, the detection rates of the RPA/CRISPR/Cas12a detection platform and qPCR detection are comparable. Both detection methods in the platform demonstrated robustness, with the LFTS method providing visible results without complex equipment, making it well-suited for on-site testing in settings where instruments may be lacking. On the other hand, the fluorescence assay, while more complex and requiring specific instruments, demonstrated higher sensitivity. Notwithstanding, the advent of portable fluorescence analyzers has mitigated such limitation, allowing for easy and rapid detection.

While our study displays the advantages of simplicity, rapidity, and sensitivity in detecting *P. aeruginosa*, it is not without significant constraints. Employing a single-gene detection strategy introduces a potential for false-negative results due to genetic variations. To expedite detection, we chose to shorten RPA amplification times, but this might miss extremely low DNA concentrations, thereby increasing the risk of false negatives and potentially reducing the sensitivity of our detection system. Furthermore, ethical constraints prevented the incorporation of clinical samples, a significant limitation to the direct clinical relevance of our findings. Employing dual or multiple genetic targets for detection might enhance accuracy, while integrating internal controls could bolster the system’s reliability. Nevertheless, the optimization and design of such a multi-target detection approach require sustained exploration and concerted effort in future research.

## Conclusion

This study has established a rapid and efficient method for the detection of *P. aeruginosa*, with broad applications in areas including the prevention and treatment of *P. aeruginosa* infection, food safety, environmental detection, and beyond. This method can significantly contribute to overall safety and the mitigation of potential risks associated with *P. aeruginosa*.

### Electronic supplementary material

Below is the link to the electronic supplementary material.

Supplementary Material 1

## Data Availability

Data and supporting materials associated with this study will be available from the corresponding author on reasonable request.

## Abbreviations

RPA: Recombinase polymerase amplification
CRISPR: Clustered regularly interspaced short palindromic repeats
Cas: CRISPR associated protein
LOD: Limit of detection
PCR: Polymerase chain reaction
