# Supplementary material for: Rapid, sensitive, and user-friendly detection of Pseudomonas aeruginosa using the RPA/CRISPR/Cas12a system
Source: BMC Infect Dis. 2024 Apr 30;24:458. doi: 10.1186/s12879-024-09348-3 (PMC11061930; doi:10.1186/s12879-024-09348-3)
Supplement: Supplementary file 1 — Supplementary Material 1 [file 12879_2024_9348_MOESM1_ESM.pdf]

Rapid, Sensitive, and User-Friendly Detection of *Pseudomonas aeruginosa* Using the RPA/CRISPR/Cas12a System

Wenjing Zhang[1]*, Hai Qu[2], Xin Wu[1], Jingjing Shi[3], Xinling Wang[3]*

Supplementary materials

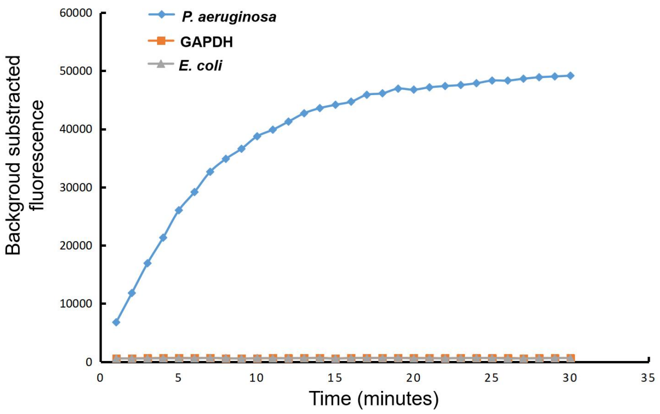

Figure S1 The fluorescence curves generated by CRISPR/Cas12a detection reaction. *P. aeruginosa*: Cas12a Protein + crRNA1 + Reporter +*P. aeruginosa* (the amplification of *P. aeruginosa* DNA using RPA); GAPDH: Cas12a Protein + crRNA1 + Reporter + GAPDH (the amplification of non - *P. aeruginosa* DNA (GAPDH gene) using PCR); *E. coli*: Cas12a Protein+ crRNA1+Reporter+E. coli (the amplification of non-*P. aeruginosa* DNA (*E. coli*) using RPA).

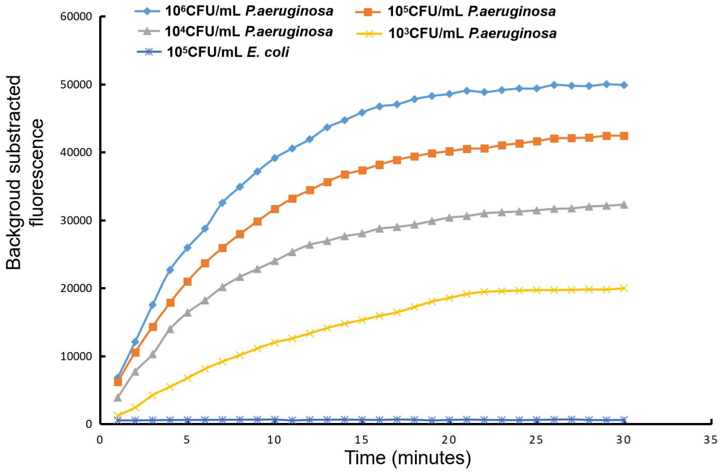

Figure S2 The fluorescence curves of different bacterial concentration generated by

CRISPR/Cas12a detection reaction. 10^6CFU/mL *P. aeruginosa*, 10^5CFU/mL *P. aeruginosa,* 10^4CFU/mL *P. aeruginosa*, and 10^3CFU/mL *P. aeruginosa* represent the fluorescence values obtained from *P. aeruginosa* at the concentration of 10^6 CFU/mL, 10^5 CFU/mL ,10^4 CFU/mL, and 10^3 CFU/mL; 10^5 CFU/mL *E. coli*: the fluorescence values obtained from *E. coli* with the concentration of 10^5 CFU/mL.

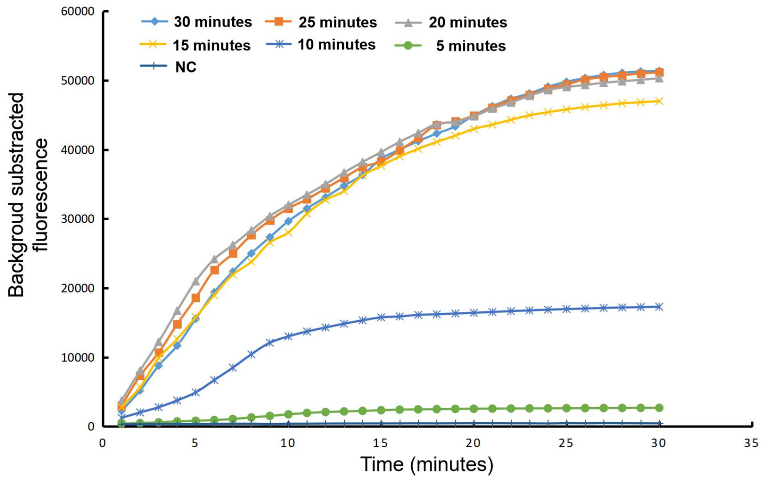

Figure S3 The fluorescence curves generated by *P. aeruginosa* DNA amplification across various time intervals detected using the CRISPR/Cas12a system. 5minutes, 10minutes, 15minutes, 20minutes, 25minutes, and 30 minutes represent the fluorescence intensities corresponding to different durations of RPA amplification (5, 10, 15, 20, 25, and 30 minutes) for *P. aeruginosa* DNA (DNA from bacterial with a concentration of 1×10^5 CFU/mL); NC: ddH_2O negative control.

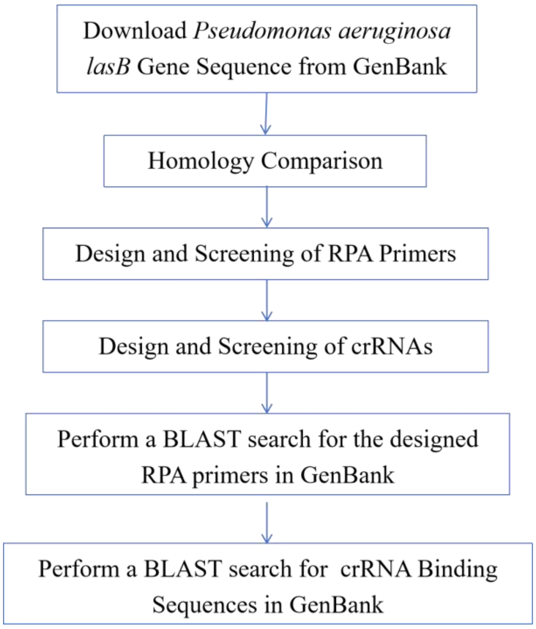

Figure S4 Flowchart of the step-by-step process used for designing RPA primers and crRNAs.

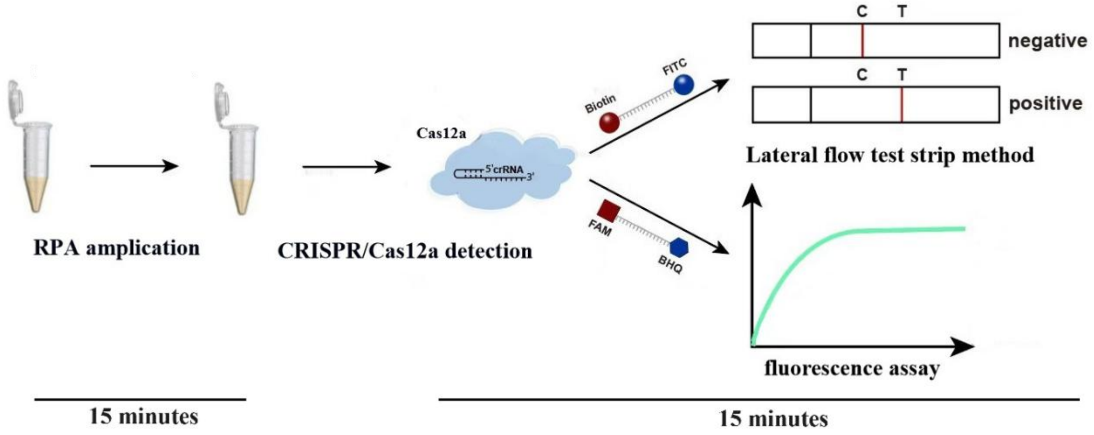

Figure S5 Illustrative diagram of the experiment process and the timeline of each step.

Table S1 Comparison between RPA/CRISPR/Cas12a detection platform and qPCR for *P. aeruginosa* detection in food samples.

Methods	Positive	Negative	Comparison between fluorescence assay and qPCR		Comparison between LFTS method and qPCR	
			Sensitivity (%)	Specificity (%)	Sensitivity (%)	Specificity (%)
Fluorescence assay	15	135	93.75	100		
LFTSmethod	13	137			81.25	100
qPCR	16	134				

Table S2 The detection results of food samples using RPA/CRISPR/Cas12a detection platform and qPCR

Sample number	Fluorescence assay		LFTS Method detection	qPCR	
	Fluorescence values	+/-	+/-	Ct values	+/-
P-1	386	-	-	No Ct	-
P-2	415	-	-	No Ct	-
P-3	432	-	-	No Ct	-
P-4	397	-	-	No Ct	-
P-5	449	-	-	No Ct	-
P-6	362	-	-	No Ct	-
P-7	395	-	-	No Ct	-
P-8	29136	+	+	25.4	+
P-9	496	-	-	No Ct	-
P-10	460	-	-	No Ct	-
P-11	397	-	-	No Ct	-
P-12	27632	+	+	26.1	+
P-13	501	-	-	No Ct	-
P-14	468	-	-	No Ct	-
P-15	372	-	-	No Ct	-
P-16	494	-	-	No Ct	-
P-17	563	-	-	No Ct	-
P-18	446	-	-	No Ct	-
P-19	523	-	-	No Ct	-
P-20	441	-	-	No Ct	-
P-21	367	-	-	No Ct	-
P-22	582	-	-	No Ct	-
P-23	8976	+	-	33.9	+
P-24	386	-	-	No Ct	-
P-25	438	-	-	No Ct	-
P-26	519	-	-	No Ct	-
P-27	388	-	-	No Ct	-
P-28	463	-	-	No Ct	-
P-29	433	-	-	No Ct	-
P-30	31057	+	+	24.5	+
P-31	446	-	-	No Ct	-
P-32	506	-	-	No Ct	-
P-33	462	-	-	No Ct	-
P-34	384	-	-	No Ct	-
P-35	426	-	-	No Ct	-
C-1	524	-	-	No Ct	-
C-2	498	-	-	No Ct	-
C-3	514	-	-	No Ct	-
C-4	531	-	-	No Ct	-

C-5	25439	+	+	26.6	+
C-6	473	-	-	No Ct	-
C-7	416	-	-	No Ct	-
C-8	498	-	-	No Ct	-
C-9	15693	+	+	28.8	+
C-10	18961	+	+	28.3	+
C-11	519	-	-	No Ct	-
C-12	318	-	-	No Ct	-
C-13	483	-	-	No Ct	-
C-14	429	-	-	No Ct	-
C-15	13846	+	+	29.3	+
C-16	466	-	-	No Ct	-
C-17	410	-	-	No Ct	-
C-18	392	-	-	No Ct	-
C-19	499	-	-	No Ct	-
C-20	21389	+	+	27.5	+
C-21	539	-	-	No Ct	-
C-22	413	-	-	No Ct	-
C-23	444	-	-	No Ct	-
C-24	514	-	-	34.9	+
C-25	21018	+	+	27.7	+
C-26	542	-	-	No Ct	-
C-27	460	-	-	No Ct	-
C-28	448	-	-	No Ct	-
C-29	419	-	-	No Ct	-
C-30	25893	+	+	26.3	+
C-31	490	-	-	No Ct	-
C-32	449	-	-	No Ct	-
C-33	517	-	-	No Ct	-
C-34	536	-	-	No Ct	-
C-35	459	-	-	No Ct	-
C-36	504	-	-	No Ct	-
C-37	19037	+	+	28.0	+
C-38	382	-	-	No Ct	-
C-39	493	-	-	No Ct	-
C-40	463	-	-	No Ct	-
C-41	491	-	-	No Ct	-
C-42	509	-	-	No Ct	-
C-43	441	-	-	No Ct	-
C-44	439	-	-	No Ct	-
C-45	29438	+	+	25.3	+
C-46	397	-	-	No Ct	-
C-47	483	-	-	No Ct	-
C-48	471	-	-	No Ct	-

C-49	528	-	-	No Ct	-
C-50	419	-	-	No Ct	-
C-51	469	-	-	No Ct	-
C-52	478	-	-	No Ct	-
C-53	460	-	-	No Ct	-
C-54	493	-	-	No Ct	-
C-55	517	-	-	No Ct	-
B-1	521	-	-	No Ct	-
B-2	446	-	-	No Ct	-
B-3	435	-	-	No Ct	-
B-4	471	-	-	No Ct	-
B-5	469	-	-	No Ct	-
B-6	454	-	-	No Ct	-
B-7	422	-	-	No Ct	-
B-8	461	-	-	No Ct	-
B-9	373	-	-	No Ct	-
B-10	361	-	-	No Ct	-
B-11	459	-	-	No Ct	-
B-12	470	-	-	No Ct	-
B-13	488	-	-	No Ct	-
B-14	390	-	-	No Ct	-
B-15	376	-	-	No Ct	-
B-16	468	-	-	No Ct	-
B-17	467	-	-	No Ct	-
B-18	449	-	-	No Ct	-
B-19	504	-	-	No Ct	-
B-20	529	-	-	No Ct	-
B-21	468	-	-	No Ct	-
B-22	441	-	-	No Ct	-
B-23	469	-	-	No Ct	-
B-24	456	-	-	No Ct	-
B-25	15654	+	+	28.9	+
B-26	387	-	-	No Ct	-
B-27	449	-	-	No Ct	-
B-28	473	-	-	No Ct	-
B-29	491	-	-	No Ct	-
B-30	392	-	-	No Ct	-
B-31	387	-	-	No Ct	-
B-32	419	-	-	No Ct	-
B-33	497	-	-	No Ct	-
B-34	466	-	-	No Ct	-
B-35	7610	+	-	34.2	+
B-36	521	-	-	No Ct	-
B-37	492	-	-	No Ct	-

B-38	476	-	-	No Ct	-
B-39	441	-	-	No Ct	-
B-40	459	-	-	No Ct	-
B-41	477	-	-	No Ct	-
B-42	469	-	-	No Ct	-
B-43	483	-	-	No Ct	-
B-44	476	-	-	No Ct	-
B-45	509	-	-	No Ct	-
B-46	446	-	-	No Ct	-
B-47	491	-	-	No Ct	-
B-48	465	-	-	No Ct	-
B-49	444	-	-	No Ct	-
B-50	542	-	-	No Ct	-
B-51	511	-	-	No Ct	-
B-52	468	-	-	No Ct	-
B-53	454	-	-	No Ct	-
B-54	392	-	-	No Ct	-
B-55	447	-	-	No Ct	-
B-56	497	-	-	No Ct	-
B-57	539	-	-	No Ct	-
B-58	423	-	-	No Ct	-
B-59	498	-	-	No Ct	-
B-60	438	-	-	No Ct	-

P-1 ~ P-35: 35 samples of processed meat products, C-1 ~ C-55: 55 samples of cold seasoned vegetable dishes, B-1 ~ B-60: 60 samples of bottled water; LFTS method: lateral flow test strip method.
